# Divergent Changes in Plant Community Composition under 3-Decade Grazing Exclusion in Continental Steppe

**DOI:** 10.1371/journal.pone.0026506

**Published:** 2011-11-02

**Authors:** Nianpeng He, Xingguo Han, Guirui Yu, Quansheng Chen

**Affiliations:** 1 Key Laboratory of Ecosystem Network Observation and Modeling, Institute of Geographic Sciences and Natural Resources Research, Chinese Academy of Sciences, Beijing, China; 2 State Key Laboratory of Vegetation and Environmental Change, Institute of Botany, Chinese Academy of Sciences, Beijing, China; Duke University, United States of America

## Abstract

An understanding of the factors controlling plant community composition will allow improved prediction of the responses of plant communities to natural and anthropogenic environmental change. Using monitoring data from 1980 to 2009, we quantified the changes in community composition in *Leymus chinensis* and *Stipa grandis* dominated grasslands in Inner Mongolia under long-term grazing-exclusion and free-grazing conditions, respectively. We demonstrated that the practice of long-term grazing exclusion has significant effects on the heterogeneity, the dominant species, and the community composition in the two grasslands. The community composition of *L. chinensis* and *S. grandis* grasslands exhibited directional changes with time under long-term grazing exclusion. Under free grazing, the *L. chinensis* community changed directionally with time, but the pattern of change was stochastic in the *S. grandis* community. We attributed the divergent responses to long-term grazing exclusion in the *S. grandis* and *L. chinensis* grasslands to litter accumulation and changes in the microenvironment after grazing exclusion, which collectively altered the growth and regeneration of the dominant species. The changes in the grazed grasslands were primarily determined by the selective feeding of sheep during long-term heavy grazing. Overall, the responses of the community composition of the Inner Mongolian grasslands to long-term grazing exclusion and heavy grazing were divergent, and depended primarily on the grassland type. Our findings provide new insights into the role of grazing in the maintenance of community structure and function and therefore have important implications for grassland management.

## Introduction

The composition of plant communities is controlled by many environmental factors and disturbances [1]–[4]. The practice of grazing is the most important land use in Inner Mongolia grasslands and has important impacts on plant community composition [5]–[7]. Therefore, understanding the changes in community composition under long-term grazing exclusion and continued grazing is very important for ecologists. This information can not only enhance our ability to predict the response of plant communities to natural and anthropogenic environmental change but also provide a scientific foundation for future grassland management.

Grasslands are typically characterized by complex disturbance regimes, including grazing, fire, and drought, each of which differs in scale, frequency, and intensity [4], [8]–[10]. The effects of disturbances on grassland vegetation can vary, and empirical evidence suggests that these disturbances often interact with each other to affect the community structure [11]–[14]. Generally, Inner Mongolian grasslands are primarily affected by complex grazing regimes [11], [15], and fire plays a relatively weak role [16], [17]. The Inner Mongolian grasslands have adapted to light grazing during their millennia-long evolution. Therefore, a marked increase in grazing intensity or a decades-long period of grazing exclusion should be somewhat equivalent to a disturbance and may influence the community structure of grasslands in the region.

Currently, improved grassland management, particularly grazing exclusion is commonly used to protect or restore the Inner Mongolian grasslands of northern China. Improved structure and function of the grasslands, such as productivity [18], community composition, C and N storage [12], [19] are expected to result from the implementation of measures aimed at grassland protection. However, there is little information about the long-term changes in plant community composition caused by the removal of large animals (grazing exclusion) from Inner Mongolia grasslands. In practice, understanding the grassland dynamics under such conditions is necessary for the sustainable management of the grasslands.

*L. chinensis* and *S. grandis* grasslands are widely distributed both in northern China and in the Eurasian steppe, and have been millenary subjected to graze by sheep and cattle. Using long-term data on plant community composition from 1980 to 2009, we analyzed changes in the community composition of *Leymus chinensis* and *Stipa grandis* dominated semiarid grasslands under grazing-exclusion and grazing-free conditions. The objectives of the study were to explore the temporal patterns in plant community dynamics over the past three decades and to assess the response of different grassland communities to long-term grazing exclusion and heavy grazing.

## Materials and Methods

### Study area

**Figure 1:**
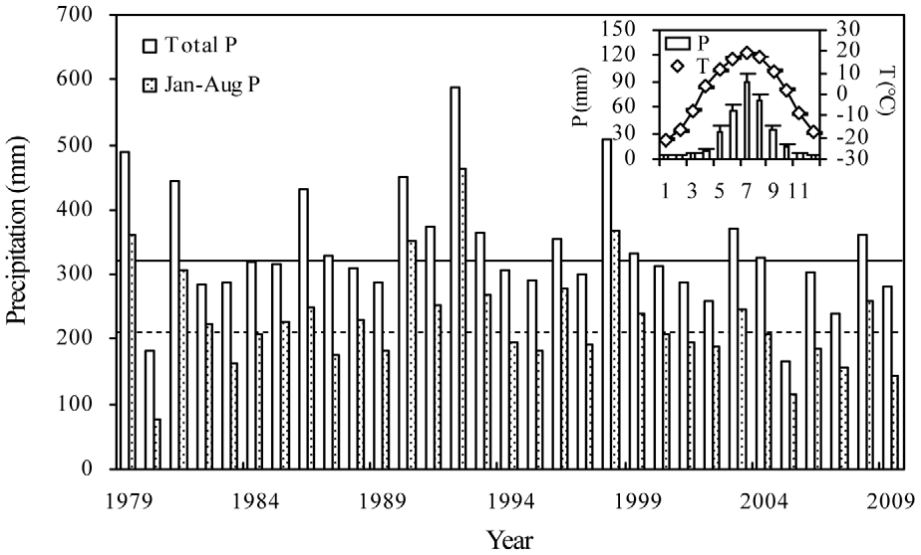
Changes in precipitation (P) and temperature (T) from 1980 to 2009 in the region of the study. The top panel shows the average monthly precipitation (mm) and temperature (°C); the solid line shows the average annual precipitation and the dashed line shows the average Jan–Aug precipitation.

The study was conducted in two semiarid grasslands in Inner Mongolia, northern China, a *S. grandis* grassland (43°32′25″N, 116°33′18″E) and a *L. chinensis* grassland (43°33′06″N, 116°40′20″E), respectively. The mean annual temperature (1980–2009) for the area is 1.1°C with mean monthly temperature ranging from −21.4°C in January to 19.0°C in July. The average annual precipitation is approximately 333.5 mm (Fig. 1). The soil is chestnut (i.e., Calcic kastanozems), equivalent to Calcic–orthic Aridisol in the US soil taxonomy classification system. The vegetation of the region consists primarily of grassland plants such as *L. chinensis*, *S. grandis*, *Cleistogenes squarrosa*, and *Koeleria cristata* [16], [17]. *S. grandis* grassland and *L. chinensis* grassland are widely distributed both in northern China and in the Eurasian steppe [16].

### Experimental plots

In 1979, the Inner Mongolia Grassland Research Station (IMGERS) established long-term experimental plots (25 hectares each) in *L. chinensis* grassland and *S. grandis* grassland. Both of which have been fenced since 1979 to prevent large-animal grazing. Simultaneously, scientists selected an adjacent area outside of each exclosures to form (fenced-grazing) paired experimental plots. These sites therefore included two-pair experimental plots (four experimental plots in all), designated as fenced *S. grandis* grassland (SG–F), grazed *S. grandis* grassland (SG–G), fenced *L. chinensis* grassland (LC–F) and grazed *L. chinensis* grassland (LC–G). The *S. grandis* and *L. chinensis* grasslands were considered to be in excellent condition and representative of lightly disturbed, climax steppe communities when the grazing exclosures were established in 1979 [17]. From 1979–2009, grazing by large animals was prevented by fences in SG–F and LC–F; the grazing grasslands (SG–G and LC–G) were grazed freely by sheep as according to the traditional grazing practices in the region. *L. chinensis* and *S. grandis* grasslands have adapted to light grazing during their millennia-long evolution. However, accompanying the economic reforms that have taken place in China since the late 1970s, the stocking rate increased from 0.51 sheep per ha in 1980 to 1.34 sheep per ha in 2000. The stocking rate was relatively stable thereafter. Therefore, SG–G and LC–G have been subjected to light degradation owing to long-term heavy grazing during the past three decades [15]. No natural or prescribed fires have occurred in these four plots since 1979. The detail characteristics of the four experimental plots are described in Table 1.

**Table 1:** Characteristics of 4 experimental plots.

Type	Treatment	Location	Soil type	Soil organic C (g kg^−1^)	Total N (g kg^−1^)	Total P (g kg^−1^)	pH	Grassland condition	Land-use history
*Stipa grandis* grassland	Fenced (SG–F)	43°32′25″N, 116°33′18″E	Chestnut†	15.91±0.46^a^ ‡ ¶	1.57±0.07^a^	0.23±0.01^b^	7.99±0.06^bc^	Excellent	Fenced and no fire since 1979
	Grazed (SG–G)	43°32′25″N, 116°31′23″E	Chestnut	13.94±0.58^b^	1.51±0.06^a^	0.26±0.01^c^	8.07±0.07^c^	Light degraded	Long-term free grazing.and no fire since 1979
*Leymus chinensis* grasslsand	Fenced (LC–F)	43°33′06″N, 116°40′20″E	Dark chestnut	17.19±0.41^a^	1.66±0.05^a^	0.29±0.01^a^	7.34±0.05^a^	Excellent	Fenced and no fire since 1979
	Grazed (LC–G)	43°32′58″N, 116°40′23″E	Dark chestnut	15.47±0.75^ab^	1.59±0.10^a^	0.29±0.01^a^	7.82±0.12^b^	Light degraded	Long-term free grazing and no fire since 1979

† The soil was chestnut (i.e., Calcic kastanozems), which is equivalent to Calcic–orthic Aridisol in the US soil taxonomy classification system.

‡ 0–10 cm soil were sampled and measured in August 2009, and values were represented as mean ± SE (n = 5).

¶ Values with same superscript letter in the same column denoted non-significant differences at *P* = 0.05 level (t-test).

### Field sampling and measurement

Within each plot, an east-west transect of 200 m×100 m was established and divided into five equal-sized replicate subplots (40 m×100 m each) [18]. Field monitoring was conducted in mid-August. In each subplot, we randomly setup one 1 m×1 m sampling quadrat during field sampling. The aboveground biomass in these 1 m×1 m quadrats was clipped at the ground level. All living vascular plants in the sample were sorted into species and dried and weighed. Because the standing biomass of these steppe communities reached its annual peak in mid-August, our estimated community biomass approximated the aboveground net primary productivity of these ecosystems [17].

In 2009, we collected soil samples from a depth of 0–10 cm using a soil sampler (diameter 4 cm), with 5 replicates in each of the four experimental plots. The samples were air-dried in a ventilated room and cleared of roots and organic debris, Air-dried soils passed through 2-mm sieve were ground for further analysis. The soil organic C content (%) was measured using a modified Mebius method [20]. Briefly, 0.5-g soil samples were digested with 5 ml of 1 N K_2_Cr_2_O_7_ and 10 ml of concentrated H_2_SO_4_ at 180°C for 5 min, followed by titration of the digests with standardized FeSO_4_. Soil organic N (%) was analyzed using the Kjeldahl acid-digestion method with an Alpkem autoanalyzer (2300 Kjeltec Analyzer Unit, FOSS, Sweden). Total P was determined by molybdenum antimony blue colorimetry. The pH of the 0–10 cm soil samples (soil∶water ratio 1∶5) was tested with a PHS-3S pH meter (Sartorius, Germany). The soil moisture was measured gravimetrically using 3 soil samples from the 0–10 cm layer at 10-day intervals in the four experimental plots in 2007. Similarly, the soil temperature was measured using sensors in 2007 in plots LC–F and LC–G.

### Data analysis

For statistical analysis, 150 non–independent samples were available from plot SG–F from 1980 through 2009. A total of 145 non–independent samples were available for plot LC–F from 1980 to 2009 because plant species data were missing for LC–F in 1986. Field sampling for the two grazed plots, SG–G and LC–G, was conducted in 1980, 1991, 1992, 1993, 1997, 1998, 1999, 2006, 2007, 2008, and 2009. Moreover, we derived the data on the community composition of the *S grandis* grassland and *L. chinensis* grassland in 1979 from the literature [17], which reported measurement made prior to the installation of the fences. In this study, the relative biomass (RB, %) of each plant species was selected to characterize the long-term changes in community composition.

To determine the small-scale compositional variability of the communities, we calculated the plant community heterogeneity at the plot scale for each year as the mean dissimilarity in the species composition of RB among the five subplots [21]. Euclidean distance (ED) was selected as our measure of heterogeneity. Therefore, community heterogeneity was designated as H_ED_ and calculated with equation 1.
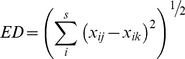
(1)where x*_ij_* is the RB of the *i*th species in the *j*th sample, x*_ik_* is the RB of the *i*th species in the *k*th sample, and S is the total number of species. Because the differences are squared, ED emphasizes the change in the abundant species from one sample to another [21].

Furthermore, a time-lag analysis of euclidean distance in community composition (designed as C_ED_) among different years was performed to determine the rate of change in community composition under different treatments over time [22], [23]. First, for comparison with the community composition in 1979, we calculated a triangular dissimilarity matrix from the species-by-time rectangular data matrix from 1979 to 2009 (the relative biomass (RB, %) of species being the variables and samples in time being the observations). Euclidean distance is also calculated as the equation 1, where *x_ij_* is the RB of the *i*_th_ species in the *j*th sample, and *x_ik_* is the RB of the *i*th species in the *k*th sample, and *S* is the total number of species. As used here, samples correspond to measurements of community composition over time. Because differences are squared, ED emphasizes change in species from one sample to the next from 1979 to 2009. Next, the Euclidean distance values are plotted against time lag for all lags below the diagonal in the triangular resemblance matrix. Linear regressions can then be calculated for Euclidean distance as a function of the square root of the time lag. The square root transformation reduces the probability that the smaller number of points at larger time lags will bias the analysis. If the regression line is significant, positive, and linear, then it implies that the assemblage in question is undergoing directional change. If the regression line is not significant or the slope is not significantly different from zero, then it implies fluctuation or stochastic variation over time. If the slope of the line is negative, then it implies species composition is converging on a community-type characteristic of one of the early sample periods. In general, the slope of the regression line indicates the rate and direction of change and the regression coefficient is a measure of signal versus noise. For example, a significant positive relationship (*P*<0.01) with a small slope and a small *r* ^2^ value suggests that directional change is occurring, but change is slow and stochastic variation between sample intervals is high. A steeper slope and larger *r* ^2^ value would indicate a stronger signal of directional change and less noise [22], [23].

To further explore the changes in plant communities, we analyzed the changes (RB, %) in 12 dominant or sub-dominant plant species. These species were *L. chinensis.*, *S. grandis*, *Agropyron cristatum*, *C squarrosa*, *K. cristata*, *Achnatherum sibiricum*, *Artemisia frigida*, *Artemisia scoparia*, *Artemisia pubescens*, *Carex korshinskyi*, *Heteropappus altaicus*, and *Poa pratensis*. We also analyzed the changes in plant functional groups (PFGs). The plant species were classified into three PFGs: perennial bunchgrasses (PB), perennial rhizome grass (PR), and others. Some researchers classified five PFGs [18]. However, we used the category designated “others” to combine perennial forbs, shrubs and semi-shrubs and annuals and biennials because their biomass was relatively minor. Moreover, we investigated the change in species richness over time in the different plots.

All data are expressed as mean ± 1 SE. The data were first checked for normality and homogeneity of variances with the Kolmogorov–Smirnov and Levene tests, respectively. Then, t-test were used to assess the differences in the soil organic C, N, P, pH, temperature, moisture, and litter among different treatments. Repeated-measure ANOVA was used to determine whether the community H_ED_ differed between the grazed and grazing exclusion plots. We also explored the influence of the total precipitation and Jan–Aug precipitation on H_ED_ and C_ED_ by performing a correlation analysis. We conducted a partial correlation analysis to evaluate the changes in the dominant and sub-dominant species and PFGs with time by controlling either the total or the Jan–Aug precipitation. All analyses were conducted using SPSS version 13.0.

## Results

The long-term grazing exclusion enhanced the soil organic C and N concentration in *S. grandis* and *L. chinensis* grasslands, but the soil pH decreased to some extent (Table 2). Moreover, the soil moisture was significantly higher in the fenced grasslands than in the grazed grasslands, but the soil temperature was significantly lower in the fenced grasslands (Table 2).

**Table 2:** Changes in soil temperature (°C) and moisture (%) during the growth period.

		Month				
	Site	5	6	7	8	9
Soil moisture (%)	SG–F†	13.90±1.28^a^ ‡	9.82±0.78^a^	7.51±0.82^a^	5.79±0.54^a^	7.59±0.93^a^
	SG–G	10.58±1.27^b^	7.77±0.57^b^	6.36±0.85^b^	5.05±0.51^b^	6.96±0.71^a^
	LC–F	17.40±0.95^a^	11.26±0.59^a^	9.94±1.79^a^	9.18±1.57^a^	9.18±1.40^a^
	LC–G	13.40±1.33^b^	8.24±0.40^b^	8.17±1.80^b^	9.10±1.79^a^	8.81±1.11^a^
Soil temperate (°C)	LC–F	10.29±0.27^a^	16.68±0.47^a^	20.75±0.28^a^	18.50±0.36^a^	13.41±0.36^a^
	LC–G	11.93±0.39^b^	19.40±0.55^b^	23.91±0.37^b^	20.35±0.48^b^	14.90±0.42^b^

† SG–F, fenced *S. grandis*; SG–G, grazed *S. grandis*; LC–F, fenced *L. chinensis*; LC–G, grazed *L. chinensis*;

‡ Data were measured in 2007, and values with the same superscript letter between fenced and grazed grasslands in same site do not differ significantly at the P = 0.05 level (t-test).

**Figure 2:**
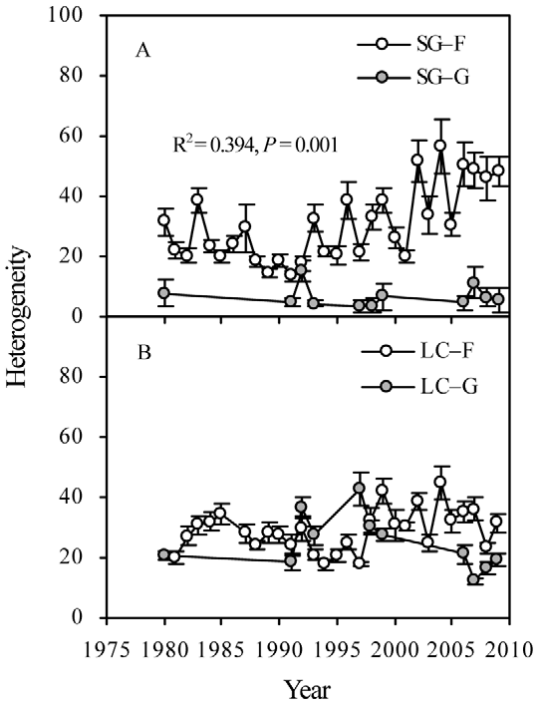
Changes in the heterogeneity (H_ED_) of the plant community with relative biomass (RB, %). SG–F, fenced *S. grandis* grassland; SG–G, grazed *S. grandis* grassland; LC–F, fenced *L. chinensis* grassland; LC–G, grazed *L. chinensis* grassland.

Community heterogeneity (H_ED_) increased significantly with time in plots SG–F (R^2^ = 0.394, *P* = 0.001) and LC–F (R^2^ = 0.145, *P* = 0.042) (Fig. 2). However, H_ED_ did not appear to change in plots SG–G and LC–G. For all four sites, H_ED_ was not significantly related to the total precipitation or the Jan–Aug precipitation (data not shown). A comparison of the paired-plot data showed that H_ED_ was significantly higher in the fenced grasslands than in the grazed grasslands (repeat-measures ANOVA, *P*<0.01).

**Figure 3:**
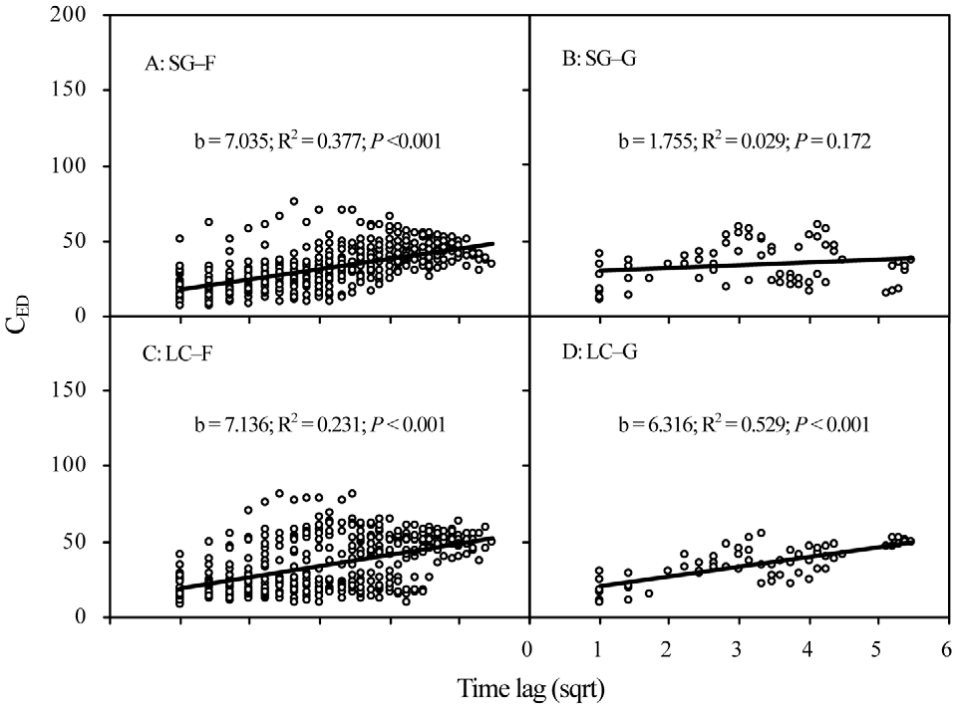
Time-lag regression analysis of compositional change of relative biomass in plant community. Regressions that were significant and positive indicated that vegetation was undergoing directional change over time. Non-significant regressions indicated that vegetation was stochastic or did not show any change. Rates of change are expressed as b ( = slope). SG–F, fenced *S. grandis* grassland; SG–G, grazed *S. grandis* grassland; LC–F, fenced *L. chinensis* grassland; LC–G, grazed *L. chinensis* grassland.

**Figure 4:**
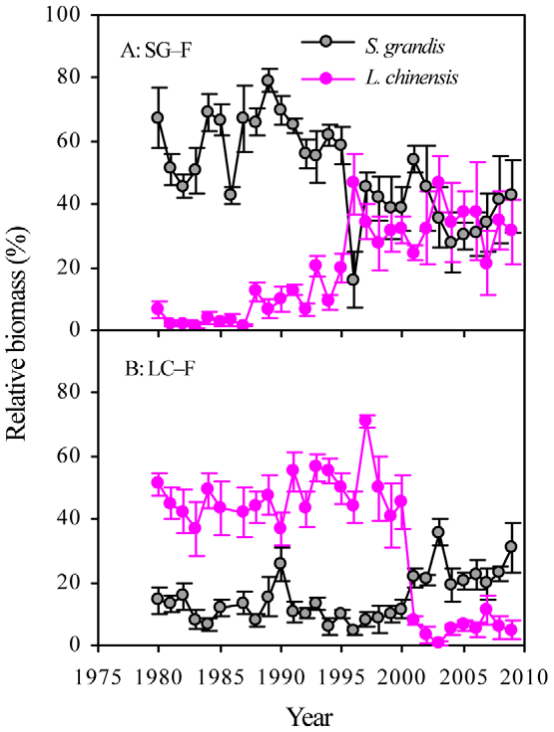
Changes in the relative biomass (RB, %) of the dominant species (*S. grandis* and *L. chinensis*) in two fenced grasslands. SG–F, fenced *S. grandis* grassland; LC–F, fenced *L. chinensis* grassland.

A time-lag analysis of C_ED_ revealed a strong linear, positive regression for C_ED_ in plots SG–F and LC–F (Fig. 3). This result indicated that strong directional changes in the community composition occurred over time. The community composition of the grazed *S. grandis* grassland fluctuated and varied stochastically over time. However, in the grazed *L. chinensis* grassland, the changes in community composition were directional over time, as shown by the time-lag analysis of C_ED_ (Fig. 3). Moreover, the value of the slope was higher in plot LC–F than in plot SG–F, indicating that the changes in community composition in the *L. chinensis* grassland were more responsive to the long-term grazing exclusion. Interestingly, changes in LC–F, as measured by the value of the slope, were also greater those in LC–G (Fig. 3). Moreover, the results showed that an unexpected apparent decrease of the dominant species (*L. chinensis*) occurred in 2001 in plot LC–F (Fig. 4).

Different time trends were found for the dominant or sub-dominant plant species in the two fenced grasslands (Table 1). In SG–F, the relative biomass (RB) of *S. granidis* (former dominant species) decreased significantly with time, while that of *L. chinensis* increased significantly with time (Fig. 4; Table 3). In contrast, the RB of *L. chinensis* (the former dominant species) in LC–F decreased significantly with time, and that of *S. grandis* increased significantly (Fig. 4; Table 3). Under grazing, there were no apparent changes in the dominant species in plot SG–F. In plot LC–F, however, *L. chinensis* decreased significantly and that of *S. grandis* increased significantly (Table 3).

**Table 3:** Changes in 12 dominant and sub-dominant species (relative biomass, %) with time, controlling for Jan–Aug precipitation (partial correlation, two-tailed test).

	*S. grandis* grassland				*L. chinensis* grassland			
	Fenced (SG–F)		Grazing (SG–G)		Fenced (LC–F)		Grazing (LC–G)	
Species	r	p	r	p	r	p	r	p
LC†	0.845‡	<0.001**	0.026	0.942^NS^	−0.689	<0.001**	−0.859	0.001**
SG	−0.642	<0.001**	0.332	0.348^NS^	0.524	0.004**	0.819	0.004**
AC	0.606	<0.001**	−0.083	0.0819^NS^	0.576	0.001**	−0.211	0.540^NS^
CS	0.300	0.114^NS^	−0.014	0.969^NS^	0.599	0.001**	0.334	0.345^NS^
KC	−0.046	0.814^NS^	−0.347	0.326^NS^	0.237	0.225^NS^	−0.844	0.002**
AS	0.191	0.320^NS^	—¶	—	0.579	0.001**	−0.690	0.027*
CK	0.591	0.001**	0.364	0.301^NS^	0.326	0.090^NS^	0.491	0.149^NS^
AF	−0.037	0.849^NS^	−0.644	0.044*	0.401	0.034*	−0.561	0.091^NS^
AS2	−0.057	0.768^NS^	−0.187	0.604^NS^	−0.282	0.599^NS^	−0.412	0.237^NS^
AP	−0.720	<0.001**	−0.800	0.005**	−0.473	0.011*	−0.838	0.002**
HA	−0.690	<0.001**	−0.800	0.005**	−0.008	0.968^NS^	−0.433	0.210^NS^
PP	0.201	0.296^NS^	—	—	−0.086	0.664^NS^	−0.281	0.431^NS^

† Abbreviations for plant species: LC, *Leymus chinensis* (Trin.) Tzvel; SG, *Stipa grandis* P. Smirn.; AC, *Agropyron cristatum* (L.) Gaertn.; CS, *Cleistogenes squarrosa* (Trin.) Keng; KC, *Koeleria cristata* (L.) Pers; AS, *Achnatherum sibiricum* (L.) Keng; CK, *Carex korshinskyi* Kom.; AF, *Artemisia frigida* Willd.; AS2, *Artemisia scoparia* Waldst et Kit; AP, *Artemisia pubescens* Ledeb.; HA, *Heteropappus altaicus* (Willd.) Novopokr.; PP, *Poa pratensis* L.

‡ NS, *P*>0.05;

*, *P*<0.05;

**, *P*<0.01.

¶ Partial correlation analysis was not conducted because the species was not present in the plot.

**Figure 5:**
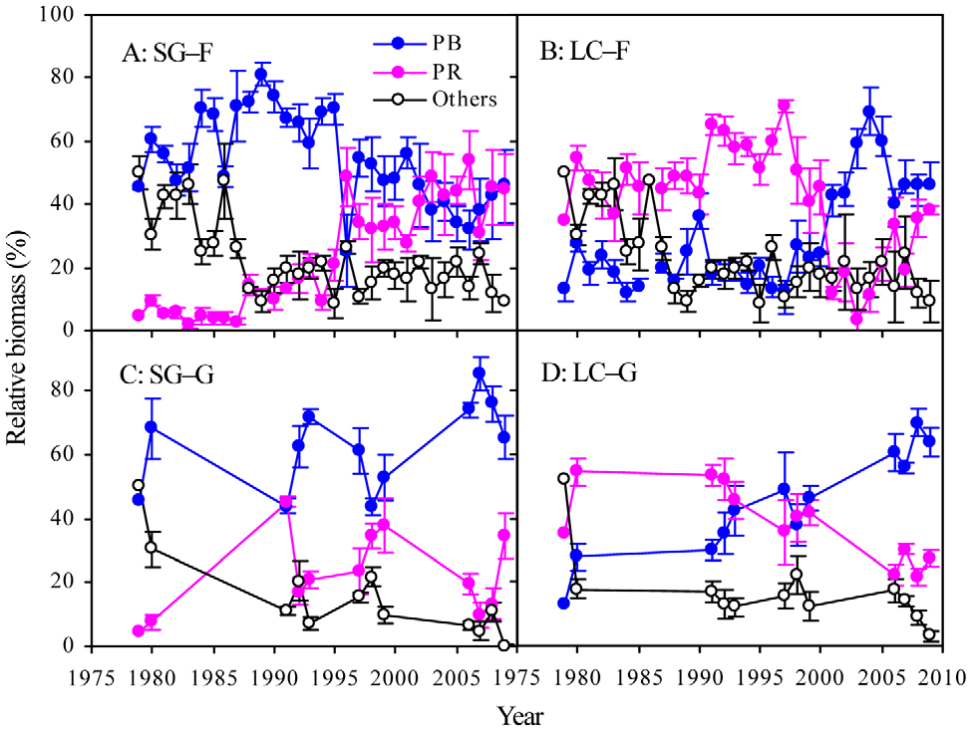
Changes in the relative biomass (%) of plant functional groups in four grasslands. The plant functional groups were classified as PB (perennial bunchgrasses), PR (perennial rhizome grass), and others. SG–F, fenced *S. grandis* grassland; SG–G, grazed *S. grandis* grassland; LC–F, fenced *L. chinensis* grassland; LC–G, grazed *L. chinensis* grassland.

**Figure 6:**
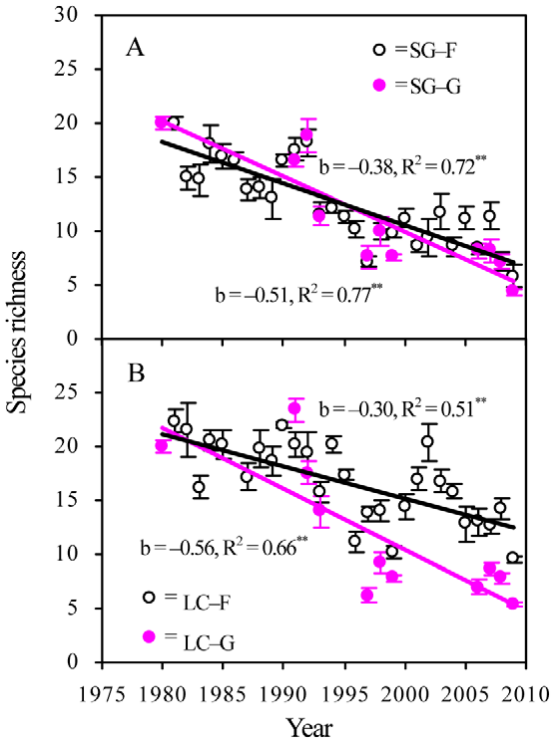
Changes in plant species richness under different treatments. SG–F, fenced *S. grandis* grassland; SG–G, grazed *S. grandis* grassland; LC–F, fenced *L. chinensis* grassland; LC–G, grazed *L. chinensis* grassland. Results of significant tests are reported as NS, *P*>0.05; *, *P*<0.05; **, *P*<0.01.

The analysis of the PFG showed that PB (the former dominant PFG) in SG–F decreased significantly with time, accompanied by a significant increase in PR (Fig. 5 and Table 3). In contrast, PR (the former dominant PFG) in LC–F decreased significantly as PB increased (Fig. 5 and Table 4). Under grazing, PR and PB did not vary with time in SG-G, but PR decreased significantly in LC–G (Fig. 5 and Table 4). Moreover, the plant species richness significantly decreased in all four plots (Fig. 6).

**Table 4:** Changes in relative biomass (RB, %) of plant functional groups (PFGs) with time, controlling for Jan–Aug precipitation (partial correlation, two-tailed test).

	*S. grandis* grasslands				*L. chinensis* grasslands			
	Fenced (SG–F)		Grazing (SG–G)		Fenced (LC–F)		Grazing (LC–G)	
PFGs†	r	p‡	r	p	r	p	r	p
PB	−0.592	0.001**	0.376	0.284^NS^	0.696	<0.001**	0.485	0.156^NS^
PR	0.895	<0.001**	0.076	0.834^NS^	−0.515	0.005**	−0.934	<0.001**

† PB, Perennial bunchgrasses; PR, Perennial rhizome grass; Others, plant species except PB and PR;

‡ NS, *P*>0.05;

*, *P*<0.05;

**, *P*<0.01.

## Discussion

The heterogeneity of the plant community in the Inner Mongolia grassland increased significantly under long-term grazing exclusion. The value of heterogeneity in the fenced grassland was higher than that in the grazed grassland. Previous studies have reported that rational grazing can enhance plant diversity and evenness in the region [24], [25]. The heterogeneity of the plant community was negatively correlated with the species richness in fenced grasslands, but no apparent trend was observed for grazed grasslands. Some studies have demonstrated that an intermediate frequency of disturbance (e.g., grazing and fire) can decrease the heterogeneity of the plant community in the North American prairie [1], [21]. Therefore, we concluded that the exclusion of large-animal grazing from these long-term fenced grasslands resulted in the observed increase in heterogeneity, which partly supported our assumption that a decades-long period of grazing exclusion should be somewhat equivalent to a strong disturbance and may influence the community structure of grasslands in the region.

The responses of community composition to long-term grazing exclusion differed in *S. grandis* grassland and in *L. chinensis* grassland. These responses were the result of the changes in biotic and abiotic factors associated with long-term grazing exclusion. After the 30-yr grazing exclusion, accumulation of litter in the surface soil in 2009 was 172.0±8.1 g m^−2^ in plot SG–F and 233.8±14.4 g m^−2^ in plot LC–F. Accompanying the increasing accumulation of litter, soil temperature was significantly lowered in the fenced grasslands than grazing grasslands (Table 2); simultaneously, the soil moisture was significantly enhanced because the fenced grasslands could retain more snow during the winter. The accumulated litter and the decreased soil temperature impeded the formation of buds or the rhizome renewal of *L. chinensis* (a perennial species with rhizomes), but their effects were far less for *S. grandis* (a perennial bunchgrass) [26]. At both sites, the removal of litter and increased soil temperatures resulting from prescribed fires will help to increase the *L. chinensis* population; simultaneously, the *S. grandis* population can be suppressed by prescribed fires because fire can directly damage the buds of *S. grandis* [27], [28]. Therefore, we assumed that changes in biotic factors, abiotic factors (especially in litter accumulation and its associated effects), and their interaction foster the divergent responses to long-term grazing exclusion.

Increased stocking rates had an apparent influence on the community composition of *L. chinensis* grasslands, but their effect on the *S. grandis* grasslands was minor. *L. chinensis* (the former dominant species) decreased significantly over time in the grazed plots. This decrease determined the directional changes in community composition (Fig. 3 and Table 3). Under natural grazing, domestic animals (primarily sheep) preferred *L. chinensis* to *S. grandis* owing to the former's higher palatability and nutrient content [29]. Therefore, under long-term heavy grazing, the changes in *L. chinensis* were mainly attributed to selective feeding by sheep [24], [25]. However, the increased stocking rates did not have the same effects on the community composition of *S. grandis* grassland because the importance of *L. chinense* in this community is minor [17]. Generally, long-term heavy grazing should affect apparently the community composition of Inner Mongolian grassland, but the type of grassland will determine the outcome.

Community composition should show abrupt changes owing to changes in the dominant species under long-term grazing exclusion in arid grassland regions. An unexpected outcome was that *L. chinensis* decreased sharply in the fenced *L. chinensis* grassland after a 21–yr grazing exclusion. A plausible explanation for the sharp decrease was that the accumulated litter decreased soil temperature decreased the availability of water from small rainfall events and affected nutrient cycles. These changes impaired the formation of buds or the rhizome renewal of *L. chinensis* and depleted the resources for growth previously accumulated in the rhizome. Some previous studies have demonstrated that many types of disturbances, including grazing, mowing, fire, plowing and harrowing, can apparently boost the formation of rhizome and rhizome buds. The enhanced rhizome and buds would cause the density and the biomass of the *L. chinensis* population to increase significantly in the following year [13], [26], [27]. Litter accumulation has multiple effects on the community structure. These effects depended on the amount of litter [11], [30], [31]. Litter has been identified as the primary mechanism for structuring grassland diversity. Richness and evenness were shown to decline with increasing litter cover [32]. The foundation provided by this information is essential for the development of general conclusion about the relationship between grazing exclusion and community composition in terrestrial ecosystems.

Further studies are required to distinguish among the divergent effects of grazing exclusion on the community structure and function in semiarid grasslands. Long-term grazing exclusion should interfere with the community composition of *L. chinensis* grassland. Therefore, long-term grazing exclusion from those high-quality grasslands should be employed cautiously. Of course, we do not deny that grazing exclusion is a beneficial approach to the restoration of degraded Inner Monoglian grassland [11], [13]. Moreover, we need to conduct further tests our plausible explanations suggested by the study by manipulating grazing, prescribed fires and other factors in the future.

The overall results of this study indicated that long-term grazing exclusion apparently influenced the heterogeneity, dominant species, and composition of the plant communities. The response of the community composition to long-term grazing exclusion differed in different grassland types. The community composition reflected sharp changes in the dominant species of the *L. chinensis* grassland under the long-term grazing exclusion. The divergent responses of the community composition can be attributed to the properties of the dominant plant species, litter accumulation, soil temperature, soil moisture, and plant–animal interaction; however, the underlying mechanism is not yet fully understood. These divergent responses and their underlying mechanisms should be important not only for empirical and theoretical research on community dynamics and for predicting the response of community composition to climate change but also for grassland management in the future.

## References

[1] McNaughton SJ (1985). Ecology of a grazing ecosystem: The Serengeti.. Ecological Monographs.

[2] Collins SL, Smith MD (2006). Scale-dependent interaction of fire and grazing on community heterogeneity in tallgrass prairie.. Ecology.

[3] Vermeire LT, Heitschmidt RK, Haferkamp MR (2008). Vegetation response to seven grazing treatments in the northern great plains.. Agriculture, ecosystems and Environment.

[4] Burns CE, Collins SL, Smith MD (2009). Plant community response to loss of large herbivores, comparing consequences in a South African and a North American grassland.. Biodiversity and Conservation.

[5] Collins SL (1987). Interaction of disturbances in tallgrass prairie: a field experiment.. Ecology.

[6] Cottenie K (2005). Integrating environmental and spatial process in ecological community dynamics.. Ecology Letters.

[7] Veen GF, Blair JM, Simth MD, Collins SL (2008). Influence of grazing and fire frequency on small-scale plant community structure and resource variability in native tall grass prairie.. Oikos.

[8] Collins SL (2000). Disturbance frequency and community stability in native tallgrass prairie.. The American Naturalist.

[9] Albertson FW, Tomanek GW (1965). Vegetation changes during a 30-year period in grassland communities near hays, Kansas.. Ecology.

[10] Hobbs RJ, Yates S, Mooney HA (2007). Long-term data reveal complex dynamics in grassland in relation to climate and disturbance.. Ecological Monographs.

[11] Li YH, Wang W, Liu ZL, Jiang S (2008). Grazing gradient versus restoration succession of *Leymus chinensis* (Trin.) Tzvel. grassland in Inner Mongolia.. Restoration Ecology.

[12] He NP, Yu Q, Wu L, Wang YS, Han XG (2008). Carbon and nitrogen store and storage potential as affected by land-use in a *Leymus chinensis* grassland of northern China.. Soil Biology & Biochemistry.

[13] Baoyin T, Li YH (2009). Can shallow plowing and harrowing facilitate restoration of *Leymus chinesnsis* grassland? Results from a 24-year monitoring program.. Rangeland Ecology & Management.

[14] Ceballos G, Davidson A, List R, Pacheco J, Manzano-Fischer P (2010). Raped decline of a grassland system and its ecological and conservation implications.. PLoS ONE.

[15] Yang H, Bai YF, Li YH, Han XG (2009). Response of plant species composition and community structure to long-term grazing in typical steppe of Inner Monoglia.. Chinese Journal of Plant Ecology.

[16] Chen ZZ, Wang SP (2000). Typical steppe ecosystem of China.

[17] Jiang S, Jiang S (1988). Setting up of the grassland ecosystem research sites and their vegetation status.. Research on Grassland Ecosystem 3.

[18] Bai YF, Han XG, Wu JG, Chen ZZ, Li LH (2004). Ecosystem stability and compensatory effects in the Inner Mongolia grassland.. Nature.

[19] Wu L, He N, Wang Y, Han X (2008). Storage and dynamics of carbon and nitrogen in soil after grazing exclusion in *Leymus chinensis* grasslands of northern China.. Journal of Environmental Quality.

[20] Nelson DW, Sommers LE (1982). Total carbon, organic carbon, and organic matter.

[21] Collins SL (1992). Fire frequency and community heterogeneity in tall grass prairie vegetation.. Ecology.

[22] Collins SL, Micheli F, Hartt L (2000). A method to determine rates and patterns of variability in ecological communities.. Oikos.

[23] Báez S, Collins SL, Lightfoot D, Koontz TL (2006). Bottom-up regulation of plant community structure in an arid land ecosystem.. Ecology.

[24] Li YH, Wang SP (1994). Response of plant and plant community to different stocking rates.. Grassland of China.

[25] Wang SP (2001). The dietary composition of fine wool sheep under different stocking rates and relationship between dietary diversity and plant diversity in Inner Mongolia steppe.. Acta Ecologica Sinica.

[26] Yang YF, Zheng HY, Li JD (1998). Comparison of age structures of the tillers in the *Leymus chinensis* population, a clonal grass species under different ecological conditions.. Acta Ecologica Sinica.

[27] Bao YJ, Li ZH, Liu ZL (2000). Influence of fire factor on *Leymus chinensis* population in Inner Mongolia steppe.. Grassland of China.

[28] Bao YJ, Zhu N, Li ZH, Liu ZL (2001). Influence of fire factor on *Stipa granids* population.. Grassland of China.

[29] Wang SP (2001). The dietary composition of fine wool sheep and plant diversity in Inner Mongolia steppe.. Acta Ecologica Sinica.

[30] Liu P, Huang JH, Sun OJ, Han XG (2010). Litter decomposition and nutrient release as affected by soil nitrogen availability and litter quality in a semiarid grassland ecosystem.. Oecologia.

[31] Carson W, Peterson C (1990). The role of litter in an old-field community: impact of litter quantity in different seasons on plant species richness and abundance.. Oecologia.

[32] Lamb EG (2008). Direct and indirect control of grassland community structure by litter, resources and biomass.. Ecology.

